# Affirming Neurodiversity within Applied Behavior Analysis

**DOI:** 10.1007/s40617-024-00907-3

**Published:** 2024-01-25

**Authors:** Sneha Kohli Mathur, Ellie Renz, Jonathan Tarbox

**Affiliations:** 1 University of Southern California, Los Angeles, CA USA; 2 University of Illinois Chicago, Chicago, IL USA; 3 University of Southern California and FirstSteps for Kids, Alhambra, CA USA

**Keywords:** neurodiversity, neurodiverse, neurodivergent, social model of disability, applied behavior analysis

## Abstract

Criticisms of applied behavior analysis (ABA) from the autistic community continue to intensify and have an appreciable impact on research, practice, and conversation in stakeholder groups. ABA providers aspire to increase quality of life for autistic people; thus, it is imperative for providers to listen with humility and openness to the population we serve. Autistic individuals have unparalleled expertise in their own lives and their own communities. The concerns raised by the autistic community cannot, morally or ethically, be swept aside. There may be a misguided and harmful tendency to devalue concerns due to the speaker’s identification as autistic or due to their difference in professional credentials. The concept of neurodiversity can help the ABA field respond to these concerns and collaborate with the largest stakeholders of our services, the autistic clients we serve. This article summarizes some of the key criticisms that autistic advocates raise concerning ABA, discusses the social model of disability and the neurodiversity paradigm, and proposes practical guidance to help the field of ABA integrate neurodiversity and thereby evolve our research and practice. By openly acknowledging the criticisms against ABA and recognizing how we can do better as a field, we believe we can take practical steps towards a profession and a society that more fully embraces inclusion.

Criticisms of applied behavior analysis (ABA) services from the autistic community continue to intensify and have an appreciable impact on research, practice, and conversation in stakeholder groups (Chapman & Bovell, 2022). ABA service providers aspire to increase quality of life for autistic people; thus, it is imperative for providers to listen with humility and openness to the population we serve. Autistic individuals have unparalleled expertise in their own lives and their own communities, whereas, regardless of the extent of education or clinical experience, a nonautistic person can never comprehensively understand the emotional, physical, and sensory experiences of being autistic. The concerns raised by the autistic community cannot, morally or ethically, be swept aside and devalued due to the speaker’s identification as autistic or due to their difference in professional credentials. The concept of neurodiversity can help the ABA field respond to these concerns, and the tenets of the neurodiversity paradigm and neurodiversity movement can be used to help the ABA field collaborate with the largest stakeholders of our services, the autistic clients we serve (Dwyer, 2022). This article summarizes some of the key criticisms that autistic self-advocates raise concerning ABA, discusses the social model of disability and the neurodiversity paradigm, and proposes practical guidance to help the field of ABA integrate concepts from the neurodiversity paradigm to address these concerns and thereby improve our research and practice. By openly acknowledging and addressing the criticisms against ABA and recognizing how we can do better as a field, we can take practical steps toward a profession and a society that more fully embraces inclusion.

Some practitioners may object to the overall message of this article by being concerned that centering client voices in the treatment process may exclude family, teacher, or clinician input. However, the ethical imperative of centering autistic voices does not entail excluding other voices. Opinions about treatment may differ between practitioner, autistic client, and client’s family and that variability is both expected and useful because each entity is viewing the situation from a different perspective and all perspectives are valuable. To this end, this article was authored by a neurodiverse team of scholars, practitioners, and advocates. This article addresses common criticisms levied against the field of ABA, but it is of course not possible to reflect the experiences of all neurodivergent people nor the practices of all ABA practitioners. Centering autistic voices means prioritizing our clients’ input in treatment planning, it does *not* mean excluding the expertise of clinicians or judgements of family members. The goal of this article is to provide practical points that the ABA research and practice communities can use for discussion and reflection, based on concerns raised by the autistic community.

## Language Matters

According to Kenny et al. (2016), although practitioners are less likely to use identity first language, feedback from self-advocates indicates a preference for identity-first language (i.e., “autistic”) over person-first language (i.e., “person with autism”). However, we acknowledge identity-first language is not a universally held preferred choice and are committed to recognizing and respecting an individual’s right to choose the terminology used to describe themselves. As such, identity-first language will be used throughout this article with the exception of any individual or group with a known desire to be referred to using person-first language.

## The Social versus Medical Model of Disability

Veneziano and Shea (2022) encourage behavior analysts to understand and study the history of disability as well as the different models of disability, and how these concepts apply to the field of ABA. Disability has historically been understood through the medical model, which conceptualizes disability as a deficit, or something to be “cured.” Thus, disability has often been approached as an inability to execute activities that are deemed “normal” by society (Thomas, 2002). For example, the *DSM-5* definition of ASD is a neurodevelopmental condition characterized by persistent deficits in social interactions, communication, and maintaining relationships. The medical model creates diagnostic labels, which influence attitudes toward bias and stigma against autistic people (Angulo-Jimenez & DeThorne, 2019). At the very heart of the medical model is the assumption that something inside the person is wrong or broken, which leads to ableism. Ableism is defined as explicit or implicit discrimination in favor of able-bodied and able-minded people (Rosqvist et al., 2020). Examples of ableism in hiring practices include favoring a nondisabled person over a disabled person, even when both are equally able to complete the tasks the job requires. An example of ableism in ABA supports for autistic people could include prioritizing particular target behaviors, for example stereotypy, because they are different from how neurotypical people commonly behave, regardless of how the behavior actually impedes functioning or how the behavior is in line with the client’s values.

According to Baglieri et al. (2011), disability discrimination should be considered a civil rights issue, because it continues to cause oppression, segregation, dehumanization, and exploitation. Discrimination against disability is not easily recognized because disability is currently understood as a health and safety issue rather than an extension of one’s identity. In our ableist society “individuals show themselves to be worthy of membership in civil society through the exercise of certain abilities. . . . Human-rights discourse will never break free from the ideology of ability until it includes disability as a defining characteristic of human beings” (Siebers, 2011, p. 178). There is clear inequity for disabled people as a minority identity and, as in other forms of societal discrimination, there is no justification for differential treatment (Baglieri et al., 2011; Siebers, 2011). These inequities often take the form of disabled people not having a say in their own treatment planning or goals, or the expectation of the disabled person to change their behaviors rather than an expectation of societal change. For example, rather than working with an organization to change the way they interview an autistic person (i.e., giving them tasks to complete that reflect their actual abilities), professionals usually focus on teaching interview skills to the disabled person, focusing on behaviors such as making eye contact or responding in a certain way to the interviewer, despite these behaviors potentially having nothing to do with the actual job itself.

The multidisciplinary field of disability studies approaches disability from a substantially different perspective from that of the medical model of disability. Disability studies is rooted in activism of people with disabilities and has influenced social and political views on disability (Baglieri et al., 2011; Siebers, 2011). Disability studies employs the social model of disability. The social model of disability reconceptualizes disability as a social, political, and cultural construct, understood in context, although still acknowledging the presence of impaired function (Baglieri et al., 2011; Siebers, 2011). From the field of disability studies also stems the cultural model of disability, which asserts that differences in understanding the concept of disability stem from different cultural contexts, and this can be seen as the understanding and treatment of disability has shifted over time periods and countries (Retief & Letšosa, 2018). Because concepts from the cultural and social models of disability are somewhat intertwined, this article will focus on the social model of disability, but we want to note the importance of the cultural model of disability. The major tenets of the social model of disability are listed below, and Table 1 reflects how to apply the social model of disability within an autism diagnosis. The social model is offered as a tool for reflection that can be used as a context to evolve practices within ABA supports for autistic people.

- Disability is a minority identity and a product of social injustice
- Disability is a socially arbitrary construct defining certain functions as normal and others as disability
- Everyone needs supports but only some people who need supports are labeled by society as disabled
- The social construction of disability is based on the dominant culture’s definition of able
- People with disabilities must be included as equal cocreators of knowledge in research and service delivery
- Disability is a civil rights and social justice issue

**Table 1:** Medical Model versus Neurodiversity Paradigm

Medical Model: DSM	Social Model: Neurodiversity Paradigm
Autism is a set of deficits to be remediated	Autism is a large and diverse set of variation in neurocognitive functioning that is, itself, just one set of variations among the infinite variation in neurocognitive functioning within our species
Persistent deficits in social communication and social interaction	Differences in perceptions, values, and approaches to social and communicative interaction
Deficits in social–emotional reciprocity	Differences in practices of expressing social–emotional connection
Deficits in nonverbal communication	Differences in use of and understanding others’ use of nonspeaking communicative behaviors
Deficits in developing, maintaining, and understanding relationships	Social dynamics that manifest in regard to neurodiversity are similar to social dynamics that manifest in regard to other forms of human diversity (e.g., diversity of ethnicity, gender, or culture). Differences in social rules and conventions and differences in preferences for some social behaviors.
Restricted, repetitive patterns of behavior, interests, or activities	Preference for structure, order, and predictability. Differences in intensity and function of some sensory stimulation.

*DSM* diagnostic criteria in the left column and examples of corresponding concepts from the social model of disability in the right column (Adapted from Mathur & Valerius, 2023)

## Defining Neurodiversity

Neurodiversity is a concept that was developed by neurodiverse individuals (Rosqvist et al., 2020), with the term itself, “neurodiversity” being coined by Australian sociologist, Judy Singer (2017). The concept of neurodiversity is informed by the social model of disability because it focuses on autistic lived experiences and how these experiences are affected by their social and cultural communities. It is important to understand and explore different ways to talk about autism because labels affect a person’s sense of identity. Language associated with a topic gives it meaning and currently the majority of discussions revolving around autism use language of neurotypical people rather than neurodivergent people (Chown, 2020). Rosqvist et al. (2020) define neurodiversity as, “perceived variations seen in cognitive, affectual, and sensory functioning differing from the majority of the general population or ‘predominant neurotype’, more usually known as the ‘neurotypical’ population” (p. 1). Neurodiversity includes, but is not limited to, autism spectrum disorder, attention deficit disorder, intellectual disabilities, and communication disorders, or a combination of such, but this article will focus on feedback from autistic individuals who have been able to communicate, verbally or in written form, their experiences with ABA. The neurodiversity paradigm has been informed by a variety of disciplines, including sociology, critical psychology, critical medical humanities, disability studies, and critical autism studies (Rosqvist et al., 2020).

Most literature on autism approaches it as a collection of deficits rather than a “diverse way of being” (Walker, 2014, p. 1). Instead, the neurodiversity paradigm approaches autism as a form of divergence rather than a deficit, with the goal of supporting researchers and practitioners to empower autistic people to maximize their potential (Rosqvist et al., 2020). Neurodiversity is conceptualized as a neurocognitive variation that, in itself, does not necessitate a negative connotation, nor does it have to imply medical pathology (Chapman, 2020). According to Chown (2020), autism can be conceptualized as a combination of neurological differences and societal oppression. In line with the social model of disability, conceptualizing autism as neurodivergence can contribute to depathologizing biopsychosocial differences perpetuated by the medical model and accompanying societal biases (Rosqvist et al., 2020).

Groups of individuals sharing a form of neurodivergence can be referred to as a neurominority rather than a group of people with disorders, the latter arguably contributing to oppression and exclusionary medical, social, and economic practices (Rosqvist et al., 2020). Despite their shared form of neurodivergence, individuals with one neurotype, such as autism, are each unique individuals (Hillary, 2020).

Autism research and practice in medicine and many other helping disciplines is currently dominated by the medical model, which reflects the neurotypical perspective. Practices based on the medical model are tailored to help individuals live independent, social, and economically productive lives, based on societal perceptions and definitions of being productive, without centralizing the neurodivergent person’s desires. Most research and practice in autism therefore seeks to cure or fix perceived deficits and minimize symptoms, which are defined by cultural and social ideals. A neurodiversity paradigm perspective counters the deficit model and instead emphasizes neurological differences as a part of one’s identity and a source of pride. According to Robertson (2010):

The neurodiversity perspective contends that living in a society designed for non-autistic people contributes to, and exacerbates, many of the daily living challenges that autistic people experience. . . . Sensory demands, social ambiguities, and information complexities are among the barriers that the modern 21st century presents to autistic people. (p. 3)

Neurodiversity demands neuroequality, or equality for all neurotypes (Rosqvist et al., 2020). In fact, it is not unreasonable to view the neurodiversity movement as one legitimate domain of the larger social justice movement. For example, Hillary (2020) discusses differences in neurocognitive communication as akin to differences in cultural approaches to communication. The author states that material from the autistic culture, including autistic autobiographies, are critical sources of information for neurotypicals to learn to be more responsive to and more humble about autistic culture. Walker (2014) argues for this perspective, stating, “The idea that there is one ‘normal’ or ‘healthy’ type of brain or mind, or one ‘right’ style of neurocognitive functioning, is a culturally constructed fiction, no more valid . . . than the idea that there is one ‘normal’ or ’right’ ethnicity, gender, or culture” (p. 1).

Although the neurodiversity paradigm is a legitimate way in which to reconceptualize disability in its own right, it is more than an academic concept. Schuck, Tagavi et al. (2022b) describe how autism interventions that currently lean more towards the medical model can be synthesized with tenets of neurodiversity within Naturalistic Developmental Behavioral Interventions (NDBIs). Unfortunately, research has also shown that behavior analysts rarely receive training on NDBIs (Hampton & Sandbank, 2022). By understanding human variation in ways that potentially help dismantle the negative aspects of the medical model of disability, putting the neurodiversity paradigm into practice may help facilitate increased empathy for people who are perceived as different, may help foster more collaborative approaches to research and treatment, foster inclusion more generally, and offers a shared language in which to talk about differences without having to pathologize them. Everyone has challenges in life, but having neurodivergent folks lead the discussion in deciding which of these challenges need support and in which ways to support them, rather than making these decisions for them, reflects the neurodiversity paradigm. In the next section of the article, we discuss how behavior analysts can put the neurodiversity paradigm into practice to help our field move forward toward greater empathy and inclusion. Table 1 illustrates how ASD is represented with the medical model in the *DSM-V-TR* (American Psychiatric Association, 2022), versus how it can be reconceptualized using the neurodiversity paradigm, a social model of disability lens (Mathur & Valerius, 2023).

## Criticisms of Applied Behavior Analysis and Practical Implications for our Field

The historical and contemporary foundations of conceptualizing the purpose of ABA as helping individuals to thrive in their lives, rather than to assimilate or behave “normally,” may serve as a rich resource for ABA practitioners to reevaluate what we do in practice today. Given the clear criticisms of ABA practice from the neurodiverse community, it seems prudent to ask ourselves if we are living up to the standards set forth by B. F. Skinner and others since, or is it possible that we are still influenced by societal ableist bias in our field’s past, that potentially carries forward to this day? Is it possible that practitioners are not today, and perhaps were never, adequately aware of the less-ableist foundations for how to conceptualize the purpose of what we do?

Practitioners of ABA may benefit from revisiting the roots of our applied science, the vast majority of which have never advocated making a human being appear “normal” as a goal of treatment. Skinner’s (1953, 1973, 1974) classic writings almost exclusively emphasized the moral imperative of creating a world that supports each human in what they care about, through the use of positive reinforcement, and eschewing aversive control. In addition, Goldiamond’s (1974) constructional approach to ABA has always emphasized beginning with a client’s strengths and focusing behavioral intervention on building upon strengths, rather than eliminating behaviors. The first edition of the classic ABA textbook by Cooper et al. (1987) provided 10 points to consider for evaluating the social significance of a behavior change target (p. 44) and 9 points to consider for prioritizing intervention targets (p. 53). Out of these 19 points, 0 recommended considering whether the behavior is different from the neurotypical population. The 19 points call on behavior analysts to consider a large variety of contextual factors, including potential for physical harm, whether the newly learned behavior will help the individual access reinforcement in their natural lives, and so on. In short, the majority of guidance in the ABA scientific literature has focused on helping individuals maximize positive reinforcement in their own lives, not on assimilation, masking, or compliance. And yet, it may be possible that a small number of sources (e.g., Lovaas, 1987) have had an outsized influence on the daily practice of ABA clinicians. It may be argued that the time has come to let go of more ableist influences within the ABA field and embrace the field’s roots of empowerment through positive reinforcement.

This section outlines common criticisms of ABA that have been voiced in academic papers, articles, and blogs. The concerns highlighted in this section represent central themes and come primarily from autistic individuals, with some accompanying nonautistic sources. The choice to center autistic voices is intentional and vital. ABA practitioners need to hear—and listen to—the passionate and critical accounts of the population we serve to fully grasp the gravity of the criticisms levied against our field. Practical implications for ABA are discussed in response to each of the criticisms and Table 2 summarizes recommendations on how clinicians may improve our practices by incorporating the core concepts of the neurodiversity paradigm.

**Table 2:** Less-Optimal ABA Practices and Potential Neurodiversity-Centered Practices

Criticism	Less-Optimal ABA Practices	Neurodiverse-Centered ABA Practices
ABA Programs Seek to Erase Autistic Identity and Encourage Masking	● Goal of making client indistinguishable from peers ● Target all stereotypy for reduction by default ● Social skills that are not relevant to client interests and preferences ● Discourage special interests	● Center client values in choosing targets ● Educate clients about neurodiversity ● Educate clients on self-acceptance ● Build treatment around client’s special interests
ABA causes or worsens mental health conditions	● Excessive escape extinction ● Ignoring assent-withdrawal ● Not responding to client’s emotional well-being ● Forced tolerance of sensory discomfort ● Resorting to coercive procedures too rapidly	● Assess for client assent and assent-withdrawal and reinforce assent withdrawal ● Monitor for harmful side-effects ● Teach self-advocacy skills ● Adopt trauma-informed care practices
ABA reduces whole human beings to individual behaviors	● Not attending to emotions ● Not inquiring about unique variables that may influence behavioral functions	● Assess emotional well-being in clients on an ongoing basis ● Collect data on client affect ● Ask clients for their input on behavioral function
Autistic voices are absent in ABA research and practice	● Treatment resources that do not include autistic input ● Addressing research topics that are the focus of neurotypical researchers	● Engage with autistic colleagues to create new treatment resources ● Invite autistic researchers as co-investigators on research ● Create autistic advisory boards
Pressuring parents to choose ABA	● Represent ABA as the default support option ● Overemphasizing possible negative outcomes if ABA is not chosen ● Overemphasizing lack of research or other concerns with non-ABA disciplines	● Consider referring some to non-ABA services if those may be a better fit ● Supportive, compassionate, zero-pressure approach ● Provide information on other evidence-based interventions and practices

Potential concerns with ABA practices (left column), corresponding traditional ABA practices (middle column), and a nonexhaustive list of examples of potential neurodiversity-centering ABA practices (right column)

## Criticism 1: Applied Behavior Analysis is Based on the Unethical Goal of Erasing Autistic Identity

Advocates argue ABA is unethical as the field seeks to erase autistic clients’ identity. The Autistic Self Advocacy Network (ASAN, n.d.) contends historical ABA practices that included conversion therapy and severe aversives, some of which continue to this day, can be neither understated nor ignored. Many autistic self-advocates draw a direct line between historical articles on conversion therapy and ABA practices today. For example, Sequenzia (2016), a nonspeaking autistic, calls ABA “autism conversion therapy” (para. 7).

Although acknowledging ABA supports for autistic people have evolved in recent decades, advocates have argued the inherent goal of ABA continues to be making autistic people appear “normal” or neurotypical, which is fundamentally unethical (Anonymous, n.d.; ASAN, n.d.). As Anonymous (n.d.) notes, a focus on socially significant behaviors, however, is not a fundamental shift in goals and orientation, as social significance is decided by neurotypicals who default to focusing on neurotypical behaviors. They argue that this modern ABA continues to ask autistics to hide their authentic selves. This perspective may be difficult for nonautistic people to see, particularly in less intrusive practices of contemporary approaches to ABA.

Lynch (2019) contends that abusive practices are often not visible to neurotypical professionals and caregivers, and often only easily understood by autistic individuals who have experienced it themselves. She describes feelings of overwhelm, sensory overload, and defeat that she states ABA can evoke as autistic children are subjected to extensive treatment that makes clear their natural way of acting is inappropriate and must be changed.

There is a concern that focusing on changing behaviors deemed different from the “norm” forces autistics to hide natural autistic traits and characteristics to blend in with neurotypical peers. Hiding one’s natural traits or behaviors is known as “masking” and can be directly taught by ABA (Rose, 2017), as well as develop naturally through an autistic individual’s life via exposure to social reinforcement and punishment. Masking and camouflaging (a synonym for masking [Bradley et al., 2021]) have been linked to serious consequences in both personal and academic accounts. Bradley et al. (2021) surveyed autistic individuals to understand the impact of camouflaging or masking. Results indicated that some dangers of masking included exhaustion, mental health issues, suicidality, inability to maintain the masking, and the individual’s autistic self not being accepted, whereas positives included being able to participate socially and strengthening coping skills and resiliency. Individuals reported not feeling the same need to camouflage after receiving a diagnosis and when they were with others that accepted and understood them. Raymaker et al. (2020) identify masking as a life stressor that may lead to autistic burnout, defined as “a syndrome conceptualized as resulting from chronic life stress and a mismatch of expectations and abilities without adequate supports. It is characterized by pervasive, long-term (typically 3+ months) exhaustion, loss of function, and reduced tolerance to stimulus” (p. 140).

Rose (2017) shared his perspective on masking and the role ABA plays in an article detailing events from his own life, stating,

Somewhere along the way growing up, I realised that i had to hide the real me away, because being different was dangerous, not fitting in drew negative attention to myself. Being me was BAD. Children put through ABA therapies now, are being taught that to behave in a Neurotypical way is GOOD. They get rewarded for behaving like a Neurotypical. . . . The constant message autistic people are given is: being autistic is BAD. Is it any wonder we kill ourselves? (ABA and Masking section)

Autistic individuals are more than 3 times as likely to attempt or complete suicide than nonautistic folks (Kõlves et al., 2021). Cassidy et al. (2018) studied camouflaging and suicidality for autistic individuals, finding a potential correlation. The research on camouflaging continues to develop, but currently provides support for the argument that an intervention teaching or encouraging masking/camouflaging may be dangerous, and this position is supported by accounts from autistics who express their lived experiences with masking (Birch, 2019; Rose, 2017; Weinstock, 2018).

### Practical Implications

Few behavior analysts participated in conversion therapy research or practice at any time and conversion therapy is universally condemned within the field of ABA today (Association for Behavior Analysis International, 2022). Nevertheless, ABA research on conversion remains part of the field’s history. Some ABA professionals may choose to support the ABA field by acting defensively in response to this criticism. However, reacting defensively will not change the historical record of research and practice in our field. More important, reacting defensively is not likely to be effective in healing the divide between the ABA profession and the autistic advocacy community. Instead, it is likely to be more effective to fully own, in public, the ethical problems of our field’s past. Practitioners in our field’s past have committed abuse (e.g., conversion therapy) and one residential school run by behavior analysts continues to use electric shock to modify the behavior of people with and without autism today (Zarcone et al., 2020). By acknowledging the abusive practices of the past and speaking out against unacceptable practices today, we can send a clear message to the autistic community that we stand for ethical and humane support for autistic people.

### A Potential Role for Values Clarification

A lack of clear and shared understanding of what constitutes behavior analytic services for autistic people, both within and outside of the ABA field, appears evident and problematic. Today, we as a field need to define ABA’s core values and explain in accessible language how these values are consistent with the values of the neurodiversity paradigm. For example, do we believe the purpose of ABA is merely to increase some behaviors and decrease other behaviors? What values does this reflect to the autistic community? Perhaps our purpose is to empower human beings to live their own chosen lives, consistent with their values and their culture? It is not the purpose of this article to prescribe specific values to the entire discipline of ABA but rather to pose questions that may help each of us orient more fully to the meaning and purpose that we share in common with the autistic community who we strive to serve.

### Erasing Autism as a Goal of Treatment

It is less common now for ABA practitioners to overtly adopt the goal of making an autistic person “indistinguishable from their normal friends” (e.g., Lovaas, 1987, p. 8), as was common in earlier literature, and contemporary ABA researchers have stated that indistinguishability as a goal lacks social validity and may be unethical in many contexts (Veneziano & Shea, 2022). However many therapy goals remain focused on changing autistic behavior to more “socially appropriate” behavior. It seems possible that the field may benefit from reorienting to a focus on improving quality of life outcomes for autistic individuals (ASAN, n.d.), and honoring the autistic characteristics and traits that the person values, while maximizing autistic strengths. Pivoting from focus on reducing autistic behaviors, to maximizing skills from a strength-based perspective may be an effective direction for ABA supports (Cosden et al., 2006). The following simple question may help direct practitioners toward this end: “Is it possible that I chose this goal / am targeting this behavior primarily because it looks different from neurotypical norms? If so, what does the client value most? Is it possible for me to adjust the focus of treatment to more fully address the client’s strengths and values?”

Because ABA supports may sometimes encourage masking, it is our responsibility to educate our autistic clients and their family on what masking is and the long-term damaging effects it can have. ABA practitioners can take practical steps with regard to masking in at least two ways. Perhaps the most obvious first place to start is to seriously question when it is necessary to target repetitive behavior for reduction. It’s possible that it is still commonplace to target stereotypy for reduction as a sort-of default approach within autism services because the common clinical lore is that the presence of stereotypy is likely to interfere with paying attention to instruction. Little or no research, of which we are aware, has demonstrated that stereotypy interferes with learning in ABA programs and autistic advocates often report the opposite, that stimming can help soothe and increase focus (Kapp et al., 2019). In addition, if a person considers engaging in repetitive behavior as part of their identity, there may be significant ethical concerns with influencing them to stop engaging in those behaviors as a default approach to supporting them. Given the strong calls from the advocacy community to not target stereotypy for reduction, a safer default strategy may be to start with the assumption that stereotypy does not need to be reduced and use positive reinforcement strategies to motivate engagement with instruction. At the level of each individual client, if reliable behavior data demonstrates that the client will not engage with instruction after multiple high-quality positive reinforcement-based strategies have been tried with high procedural integrity and after examination of the MOs or altering the instructional target, then the treatment team could consult the client and the family to consider the least intrusive procedures for decreasing repetitive behavior during instructional times. And even then, the team might consider encouraging stimming outside of instructional times, as research has shown that encouraging stereotypy in one setting is not likely to increase stereotypy in other settings (Charlop et al., 1990).

Second, ABA practitioners may choose to play an active role in helping clients cope with masking requirements that are imposed on them from the world outside of the ABA program. ABA practitioners could consider educating clients about recuperation strategies for autistic burnout that have been identified by the advocacy community. Such activities include supporting the autistic individual to find time to live genuinely autistically, including unmasking, finding individuals that are supportive and accepting, time isolated from others, and the reduction of activities (Raymaker et al., 2020).

## Criticism 2: Applied Behavior Analysis Overrelies on Compliance and Causes Long-Term Negative Impacts for Autistic People

Many autistics have raised concerns about potential long-term impacts of ABA practices that emphasize compliance, withhold rewards, and use physical prompting against the individual’s will (ASAN, n.d.; George, n.d.-b). Speaking of their own experiences as an autistic youth, Sparrow (2016) writes,

Therapy should make your child better, not traumatize them, possibly for many years, potentially for the rest of their life. A therapist might tell you that “a little crying” is a normal thing, but I was once an Autistic child and I can tell you that being pushed repeatedly to the point of tears with zero sense of personal power and knowing that the only way to get the repeated torment to end was to comply with everything that was asked of me, no matter how painful, no matter how uneasy it made me feel, no matter how unreasonable the request seemed, knowing that I had no way out of a repeat of the torment again and again for what felt like it would be the rest of my life was traumatizing to such a degree that I still carry emotional scars decades later. (para. 18)

Likewise, Lynch (2019) criticizes ABA procedures that do not allow the child to withdraw their participation during therapy, noting that children learn compliance will cause the distressing situation to stop and are likely left vulnerable to abuse due to participation in a therapy that extinguishes self-advocacy. This may be particularly concerning when therapy that extinguishes self-advocacy lasts up to 40 hr per week for years (Lynch, 2019; Sparrow, 2016; George, n.d.-a). Sandoval-Norton and Shedky (2019) argue that ABA can lead to learned helplessness, lowered self-esteem, anxiety, and overcompliance. George (n.d.-b) describes autistic adults exposed to ABA as having life-long difficulties with issues of consent and compliance. Given disabled/autistic individuals’ increased risk of abuse/assault (Brown-Lavoie et al., 2014; Weiss & Fardella, 2018), it follows that overemphasizing compliance in ABA therapy may lead to increased vulnerability throughout one’s lifetime.

Advocates further criticize ABA for a direct role in causing or worsening mental health conditions. They purport that the use of ABA with autistic individuals leads to increased rates of posttraumatic stress disorder (PTSD), referencing a research study by Kupferstein (2018), which found individuals treated with ABA in childhood showed higher posttraumatic stress symptoms than those not treated with the intervention. This study, although preliminary, provides initial evidence for the potential negative effects of ABA while providing a record of the reflections of autistic adults that have experienced the intervention first-hand.

### Practical Implications

Assessing and honoring client assent throughout the treatment process would eliminate the ABA field’s reliance on escape extinction and compliance training (Breaux & Smith, 2023). When a client indicates that a demand is nonpreferred, either through “socially appropriate” functional communication, or through engaging in escape-maintained “challenging behaviors,” we may benefit from using that as a learning opportunity for us as practitioners and researchers. The client is telling us in that moment that they are not comfortable with what we are asking them to experience and merely persisting with the demand until they comply may be building an overly rigid repertoire of compliance with adult demands. We do not want our neurotypical children to arbitrarily comply with any demand an adult gives them, even when they feel uncomfortable, so why would we want to unintentionally build this repertoire in our autistic clients?

It is clinically important to help our clients build more adaptive topographies of self-advocacy and assent-withdrawal, other than engaging in what the neurotypical society would label as inappropriate or maladaptive behavior. It is fortunate that substantial research has been published on procedures for managing escape-maintained challenging behavior without extinction and the research continues to develop (see Chazin et al., 2022, for a systematic review of 39 studies). By basing our procedures on the assumption that a client has a fundamental right to choose to assent to treatment in the moment, we may be more likely to ensure that we expose clients to challenging situations when they themselves value it, or at a minimum, when there is sufficient beneficial positive reinforcement that the client finds such challenges “worth it.”

Neurodiversity advocates have raised a variety of concerns around the use of contingent reinforcers in ABA and these concerns require a subtle and thoughtful response from our field. On the one hand, it is not unethical to withhold positive reinforcers contingent on behavior. Money is withheld until a worker completes their work, course grades are withheld until the student completes course assignments, and so on. It is not the use of contingent rewards per se that is problematic. One specific concern that neurodiversity self-advocates raise seems to point to the manner in which reinforcers are withheld. The BACB *Ethics Code* (section 2.15) dictates that we assess for the potential harmful side effects of intervention procedures (Behavior Analyst Certification Board, 2020) and neurodiversity advocates are telling us that the ways in which reinforcers are sometimes used bring about harmful side effects. We might do well to center the voices and concerns of our clients and be judicious about how we choose which reinforcers to withhold. Of course, this decision will need to be made in unique ways with each individual client and context but some relatively straightforward examples include not withholding access to anything that can generally be described as safety or comfort. For example, a child’s security blanket, or access to comfort from their mother when they are upset, or access to other comfort stimuli when they are feeling anxious, might not be good choices for stimuli to withhold for use as contingent positive reinforcers (Rodriguez et al., 2023).

Finally, adopting practices from trauma-informed care (TIC) into ABA services may be helpful in addressing the concerns described above. Rajaraman et al. (2022) contends most ABA settings with individuals at-risk for exposure to trauma are well-positioned to incorporate TIC into their practice, as barriers are surmountable and existing behavioral analytic principles and ethics are congruent with TIC. The components of TIC are to “(a) acknowledge trauma and its potential impact, (b) ensure safety and trust, (c) promote choice and shared governance, and (d) emphasize skill building” (Rajaraman et al., 2022, p. 44). Rajaraman et al. provide guidance in each area for ABA practitioners, noting that the last of these, emphasizing skill building, is a core attribute and strength of ABA. They recommend multiple strategies and considerations, including on-going discussions and agreement on the treatment plan; considering the threat to emotional safety presented by restraint, regardless of physical safety factors; providing the choice of nonengagement in therapy; conceptualizing behaviors in the context of previous trauma; and considering the role of previous trauma in client reactions to specific interventions and avoiding identified techniques to preclude retraumatization (2022). Rajaraman et al. (2022) notes the potential positive effects of TIC in ABA, notably avoidance of retraumatization or traumatization and the ability to address concerns in the autistic community about the potentially traumatizing nature of ABA, while identifying the need for short-term and long-term research on adaptive functioning and mental health issues to further address these concerns.

## Criticism 3: Applied Behavior Analysis Reduces Autistic People to Overt Behaviors

ABA is sometimes viewed as reductionistic, focusing on a small number of overt behaviors at the exclusion of considering the individual as a whole person with a variety of needs. As expressed by ASAN (n.d.),

ABA classifies all behavior of an autistic person into four functions: to gain attention, to gain access to a desired item, to escape a demand or task, and to gain or escape sensory input. . . .

This view of behavior portrays an autistic person less as a human and more as a machine that processes inputs into outputs. It ignores complex internal reasons someone may “act out.” It denies autistic people the human dignity and compassion that other people experiencing pain or discomfort receive. Reducing behavior down to four functions allows intervention practitioners, educators, and even parents and caregivers to stop any inquiry as to why an autistic person is actually behaving a certain way. (Dehumanization of Autistic People section)

Gardiner (2017) found respondents (autistic individuals and individuals with related disabilities) valued the following in behavioral treatments: “promoting positive outcomes, preventing harm to people with disabilities, protecting people’s autonomy, advocating for inclusion, being sensitive to people’s past trauma, and supporting cultural competency” (p. 1).

### Practical Implications

The science of ABA is defined as a science that focuses on functional relations between behavior and environment and it seems possible that we may sometimes focus on overt behavior too rigidly when interacting with the human beings who we serve. Although they are not easily directly measured, private events such as thoughts, emotions, and physiological states are relevant because our clients are whole human beings. Private events have been a part of our science since the earliest articulations of Skinner’s radical behaviorism (Skinner, 1945), and more contemporary behavior analysts have called for greater attention to them (Friman et al., 1998). Our clients are not merely the sum of their overt behaviors, nor are we, and the autistic community is telling the field of ABA that our procedures and the way we talk about them sometimes gives the impression that all we care about is overt behavior. Research has shown that autistic children experience significant unmet mental health-care needs (Menezes, 2021). One way that we can show the autistic community that we care about them as whole humans, while also better supporting our clients, would be to increase our efforts at educating our clients and their families about unmet mental health-care needs in autism and to take action to connect our clients with funded mental health-care services.

An additional way in which we can show the autistic community that we genuinely care about them as whole human beings is to further base what we do on compassion. A recent article by Rodriguez et al. (2023) provides simple guiding principles for basing ABA practices on a foundation of compassion, which necessarily involves empathizing with others and taking action to alleviate their suffering, including suffering which we as practitioners may be causing. Taylor et al. (2019) provide 18 specific skills for practitioners to engage in empathetic and compassionate interactions with clients’ caregivers. All of these skills can be role-played and trained, like any other staff skill. In addition, a recent article evaluated a specific procedure, deemed “kind extinction,” for validating client emotions during extinction (Tarbox et al., 2023). More replication is needed, but the initial study showed that autistic clients can be given emotional comfort and support in their own preferred format, contingent on challenging behavior, although simultaneously implementing extinction functionally.

Some neurodiversity advocates have expressed concern that compassion implies that autistic people should be pitied and that compassion could be implemented from a saviorist standpoint (Neuroclastic, n.d.). We agree that it is important to always be thoughtful about how attempts at improving ABA may be unintentionally implemented in ways that do harm and therefore it is always important to hold closely to a behavioral functional understanding of what we do. Compassion, as defined behaviorally, means acknowledging suffering wherever we see it (in autistic people or anyone else), empathizing with it, and taking overt action to ameliorate it *in the way in which the client wants to be treated* (Taylor et al., 2019). A radical approach to compassion calls for compassion for all human beings, so applying this approach to autistic folks simply means that autistic people are equally worthy of being treated with compassion as neurotypical folks because autistic people are whole human beings (Rodriguez et al., 2023).

Because the “four functions” can be perceived as invalidating, it may be worth considering how we might augment our functional assessments with indirect data from the people we serve. For example, autistics often report that stereotypy serves a self-soothing and self-regulating function (Kapp et al., 2019; Sparrow, 2021). In behavioral jargon, we might consider these behaviors automatically negatively reinforcing if they help decrease an aversive state. We may consider asking our clients or their parents why they engage in automatically reinforced behavior, in order to demonstrate a humble curiosity to hear the individual’s perspective and a willingness to consider how the behavior may be adaptive.

In the case of escape-maintained behavior, we may address the autistic community’s concerns by more carefully considering—and asking the autistic person themselves, when possible—*why* the demands are aversive. Are the demands too difficult, too boring, too repetitive, etc.? If clients do not possess the verbal skills to self-report their emotions, behavior analysts can directly measure indices of emotion, with evidence-based procedures such as those described by Reid (2016). In sum, it may be worth considering that we are not done with ascertaining function when we identify one of the four functions. We may then want to further enquire as to what about the environment, for this particular human, in this particular context, is creating the establishing operation for that behavior occurring for that function.

## Criticism 4: Autistic Voices are Absent in ABA Research and Practice

Autistic self-advocates have criticized ABA for our almost complete lack of autistic input in ABA research and practice that attempts to serve the autistic community. Inclusion is a moral and ethical imperative that demands that autistic people have a voice in the research and practice that is aimed at helping the community.

### Practical Implications

We as a field can choose to make it a core value to fully include the autistic community in ABA research and practice that focuses on serving autistic people, by centering and amplifying autistic voices in all ABA spaces that are related to autism.

#### Centering Autistic Voices in Research

Guidelines have already been published for including autistic people in research in a manner that is affirming and empowering (Gowen et al., 2019) and these guidelines should become a standard tool researchers use when planning autism research. Research should be a collaborative process in which the researcher and participant together steer the study to share how the participant experiences the world (Berryman et al., 2013). Practical steps that ABA researchers can begin to take immediately include inviting autistic colleagues to collaborate as full co-investigators in ABA research, constructing research review committees that center autistic voices, and inviting autistics to contribute to the peer-review process.

One potential barrier to centering autistic voices in ABA research is that young children often lack the skills to meaningfully collaborate on a research team. One option may be to include autistic individuals on the research team who received services in the past similar to the procedures being researched today. Given that intensive ABA has been available in the United States for several decades, there are thousands of autistic adults who received services in the past and are able to recall what their experiences were like and these recollections should be valued.

#### Centering Autistic Voices in ABA Graduate Programs

Including more autistic students in graduate programs in ABA would help center and amplify autistic voices in the graduate training process, in addition to training the next generation of autistic professors in ABA. Including more autistic students in graduate programs may require intentional efforts on the part of the program and faculty, including outreach efforts to neurodiverse communities, explicit training for admissions faculty on neurodiversity and on cultural humility, creating neurodiversity-affirming classroom experiences, advocating for accommodations, and valuing diverse forms of class engagement. A second way to center autistic voices in higher education in ABA is to include autistic voices in coursework. This can be achieved by inviting autistic guest speakers to discuss their lived experiences. Whenever possible, autistic guest speakers should be compensated for their time and for sharing vulnerable and sometimes traumatic experiences with us. Another way to center autistic voices is to assign autobiographical books written by autistic authors. With the advent of social media, there are also several informal platforms in which neurodivergent folks can discuss their experiences (e.g., Facebook, Instagram, TikTok, Twitter (now called X), blog posts). These platforms should be explored in order to learn directly from autistic individuals.

#### Centering Autistic Voices in ABA Agencies

It may be useful for organizations to spend some time and effort clarifying the organization’s values and purpose both at the leadership level and in larger staff meetings (Flaxman et al., 2013). Once clear organizational values are identified, it will be beneficial to have a frank discussion of how honoring neurodiversity helps the organization move toward or away from those values. If an organization is serious about affirming neurodiversity, how might the organization enact those values at the organizational level? It may be worth considering creating a neurodiversity advisory board or hiring neurodiversity consultants. The advisory board or consultant may be tasked with evaluating organizational practices and making recommendations for how the organization can produce internal change that helps move toward affirming neurodiversity.

To avoid tokenism, it is important to bring in experts who are not simply neurodivergent themselves (although that is critical) but also have experience and expertise in organizational change that affirms neurodiversity. It is also worth noting that neurodivergent individuals who are brought in from outside the organization and asked to consult on difficult and often traumatic topics must be compensated for their labor, especially considering how chronically underemployed autistic adults are (Shattuck et al., 2012). A manual for helping neurotypicals successfully and respectfully include autistic consultants, including advice on compensation and other issues, is freely available (Nicolaidis et al., 2019).

There is likely no other single practice that will have a larger long-term benefit than recruiting, hiring, retaining, and promoting autistic employees. Specific additional efforts may need to be made to create connections with neurodivergent social groups, professional associations, clubs, and so on, in order to create a recruiting pipeline (Griffiths et al., 2021). After autistic employees are hired, organizations may need to take specific actions toward retaining them. Neurodivergent workers often have different needs for support or accommodations and if we are serious about centering autistic voices in our agencies then we may need to take these needs and accommodations seriously. We may need to confront and create some flexibility around some of our long-held biases around what we consider *appropriate social behavior* at work or what we consider to be *professionalism.*

## Criticism 5: Professionals Pressure Parents into Only Considering ABA

Some advocates believe that ABA providers prey on parents in times of fear, confusion, and uncertainty. The societal stigma of an autism diagnosis can instill strong emotions in parents, leaving them in a vulnerable position. Jenicanadaylivecom (2019), the mother of an autistic child, details the failings of various systems and lack of appropriate services for her autistic and blind son. She desired respectful, empowering interventions, and the professionals reactions to her desires led her to feel that the professionals blamed her and her choice of interventions for her child’s struggles. She explains professionals pressured her and leveraged her fear to compel her to allow treatments for her son that went against her beliefs, including a residential ABA facility that used techniques she describes as traumatizing and “torture” (Jenicanadaylivecom, 2019, para. 22). Her son’s behaviors increased, in part, she believed, because he refused to give in to demands for compliance. Rosa (2020) shares her panicked feelings after her child’s autism diagnosis leading her to consult with those deemed experts in the field, complying with the recommendation of ABA, a choice she states she would not have made after learning from autistic individuals. Both situations describe individuals positioning themselves as experts and exerting coercive means to convince the parent their child required ABA therapy. The perception that parents are sometimes agreeing to intensive levels of therapy under duress or that they are consenting to procedures that they are not actually comfortable with is concerning for our profession.

### Practical Implications

A couple of decades ago, ABA was the only treatment approach for autistic people with a substantial amount of research support published in scientific journals. Much research has been published in recent years that supports other approaches that include components of ABA and/or are complementary to ABA, especially those described as naturalistic developmental behavioral interventions, for example the Early Start Denver Model and Joint Attention, Symbolic Play, Engagement and Regulation intervention (JASPER; Schreibman et al., 2015). Research has shown that many ABA professionals are not aware of this research (Hampton & Sandbank, 2022) and it is possible that many still present ABA as the only evidence-based treatment and therefore the only option for parents of newly diagnosed children to choose. The BACB ethics specifically notes the importance of behavior analysts “Acknowledging that personal choice in service delivery is important by providing clients and stakeholders with needed information to make informed choices about services” (BACB, 2020, p. 4). From this standpoint, it is our ethical responsibility to educate ourselves about NDBIs and other evidence-based options, and to educate our potential clients to make the most informed decisions.

Parent training approaches inside of ABA may benefit from being built on a foundation that is informed by the neurodiversity paradigm. Rather than first focusing parent training efforts on behaviors that need to be increased and decreased, we could consider first teaching parents that their child is a unique, precious human being, who may have a brain that is different from many others, in ways that are both challenging and exciting. Perhaps the purpose of parent training should be to help parents see the world through their child’s eyes and empower their child to thrive in a world that is in many ways a mismatch between their neurotype and the way the world is socially structured. In other words, we should start by teaching parents that our and their job is to understand their child’s unique perspective and values, and then build therapeutic learning opportunities around those unique values and perspectives. By reconceptualizing the diagnosis itself, parents may feel less distressed and more inclined to find the appropriate accommodations for their child (Brown et al., 2021).

Table 2 lists the concerns discussed thus far, some potential practices in ABA that may not be useful in addressing these concerns, and some alternative practices that we could consider adopting to a greater extent than we currently have. The table is not meant to be either exhaustive or prescriptive. It is also particularly important to note that this article is not suggesting that any particular ABA provider is engaging in any or all of the less-useful practices in the middle column of Table 2. Rather, the practices listed in this table are simply examples that some ABA providers and researchers may find useful as points of reflection and discussion. Of course, the most useful practices of moving towards alignment with the neurodiversity paradigm with any particular client or within any particular organization are going to be multifaceted, complex, and contextually dependent.

## Discussion

One potential objection to the current article is that it does not represent the full range of concepts, values, and concerns held by the neurodiversity community. In particular, some neurodiversity advocates may argue that the criticisms of ABA that are discussed in this article do not go far enough. In an article-length treatment, it is not possible to give a comprehensive discussion of the entire topic, nor did we attempt to do so. The intended scope of the current article was an inclusive-enough representation of the issues for ABA researchers and practitioners to take some tangible first steps toward affirming neurodiversity in daily research and practice. Many more articles and books will likely be needed to extend the current discussion, as well as taking the discussion in other directions that have not been touched on here. Thus we hope this article will continue a much-needed conversation in the ABA literature by building upon work by Veneziano and Shea (2022) and Schuck, Dwyer et al. (2022a).

One direction for future research would be to evaluate commonly practiced ABA procedures in terms of the degree to which the procedures affirm neurodiversity and/or center the voices and values of the people being served. Several possible research avenues could be pursued in this direction. For example, further qualitative research could be conducted in which autistic adults who have received ABA services could be interviewed and common themes that emerge could be identified and yield directions for how ABA procedures can be improved. In addition, large-scale quantitative survey research needs to be conducted across large populations of people who have received ABA services, across a diverse variety of regions and cultures, with intersectionalities of a person’s identity being taken into consideration during treatment planning.

Smaller-scale studies could also evaluate specific modifications to ABA procedures for their effects on social validity from the perspective of individual autistic clients (Schuck, Dwyer et al., 2022a). Finally, as recommended by Parsons et al. (2012), identifying and validating indices of happiness versus unhappiness during behavioral programs should be studied, especially with those who are limited vocally.

## Conclusion

The field of behavior analysis is at a crossroads. How we respond to the criticisms levied against our field will define us as much, if not more, than the criticisms themselves. Criticisms against ABA include the historical underpinnings of what ABA was used for, such as conversion therapy, the use of aversive techniques, an emphasis on teaching neurotypical social skills and behaviors, exploiting parental learned helplessness, a lack of emphasis on teaching self-advocacy skills, trying to replace sensory soothing behaviors rather than understanding them, a lack of collaboration with other evidence based support therapies, overemphasis on compliance training, and long-term negative mental health impacts.

Although we cannot change the history of our field, we must acknowledge the pain our field has caused to some, and we can certainly commit to doing better. First and foremost, whether we agree with the criticisms or not, we must listen to the biggest stakeholders in our services, our autistic clients. We must ***believe*** them when they tell us that their experiences were traumatic. We must center their voices, understand their concerns, and include them in the solutions that we offer. We can do so by viewing autism through a social model lens rather than a medical model lens. Rather than thinking of ourselves as the sole experts, we can reconceptualize our treatment approach as a collaborative process between experts in behavior (us) and experts in autism (our clients). With this conceptual change, we can recognize that when using the medical model of disability, no matter how well-meaning we are, we may inadvertently encourage neurotypical behaviors. Including autistic clients in service planning and delivery, including their judgment as to whether they need or desire these supports, will allow ABA practices to improve desired quality of life outcomes and move us towards social justice for a historically oppressed neurominority.

It is imperative that we find a common goal, and that both sides understand and trust that the goal is the same—to optimize the lives of an autistic individual without sacrificing their unique autistic self, and sometimes this means that an autistic person does not require any treatment. As ABA practitioners, we must engage in continuous self-reflection. We must ask ourselves, why are we choosing that particular goal/intervention? Is it for our client’s benefit or the comfort of neurotypicals in alignment with sociocultural and behavioral expectations? We must redouble our efforts to involve our clients in treatment planning and progress monitoring (i.e. what do **they** want to work on; which strategies/approaches feel best to **them**).

Incorporating the tenets of the neurodiversity paradigm into ABA will involve recognizing that ABA is not the only support that can help autistic folks, and whenever possible ABA practitioners should engage in cross-disciplinary collaboration in order to best support our client’s diverse needs. Autism is just one part of a person’s identity. We can move forward by ensuring that other factors are being considered (e.g., ethnicity, gender identity, culture, race, class, physical ability, immigration/refugee experiences, language, education)

Finally, ABA practitioners must recognize that many in the autistic community may have significant difficulty trusting a provider, ABA or otherwise. We will thereby need to recognize the very real trauma endured in therapeutic settings, medical systems, and psychiatric systems. This cannot be easily undone in an individual or a community. Further, we must acknowledge the history of ABA and its significance. Many practices may not continue today, but they *still happened* in the past and that cannot be ignored. We believe that the goals and values of advocates of the neurodiversity paradigm and ABA providers have far more in common with one another than in difference. We believe that the future holds great promise for the field of ABA to grow and evolve toward greater inclusion, compassion, and effectiveness by centering neurodiversity in all that we do in supporting the autistic community.

## Data Availability

Data sharing is not applicable to this article because no datasets were generated or analyzed during the current study.
